# Reducing Cognitive Load and Improving Warfighter Problem Solving With Intelligent Virtual Assistants

**DOI:** 10.3389/fpsyg.2020.554706

**Published:** 2020-11-17

**Authors:** Celso M. de Melo, Kangsoo Kim, Nahal Norouzi, Gerd Bruder, Gregory Welch

**Affiliations:** ^1^Computational and Information Sciences, CCDC US Army Research Laboratory, Playa Vista, CA, United States; ^2^College of Nursing, University of Central Florida, Orlando, FL, United States; ^3^Institute for Simulation and Training, University of Central Florida, Orlando, FL, United States; ^4^Department of Computer Science, University of Central Florida, Orlando, FL, United States

**Keywords:** intelligent virtual assistant, collaboration, cognitive load, embodiment, augmented reality

## Abstract

Recent times have seen increasing interest in conversational assistants (e.g., Amazon Alexa) designed to help users in their daily tasks. In military settings, it is critical to design assistants that are, simultaneously, helpful and able to minimize the user’s cognitive load. Here, we show that embodiment plays a key role in achieving that goal. We present an experiment where participants engaged in an augmented reality version of the relatively well-known desert survival task. Participants were paired with a voice assistant, an embodied assistant, or no assistant. The assistants made suggestions verbally throughout the task, whereas the embodied assistant further used gestures and emotion to communicate with the user. Our results indicate that both assistant conditions led to higher performance over the no assistant condition, but the embodied assistant achieved this with less cognitive burden on the decision maker than the voice assistant, which is a novel contribution. We discuss implications for the design of intelligent collaborative systems for the warfighter.

## Introduction

In the near future, humans will be increasingly expected to team up with artificially intelligent (AI) non-human partners to accomplish organizational objectives (Davenport and Harris, 2005; Bohannon, 2014). This vision is motivated by rapid progress in AI technology that supports a growing range of applications, such as self-driving vehicles, automation of mundane and dangerous tasks, processing large amounts of data at superhuman speeds, sensing the environment in ways that humans cannot (e.g., infrared), and so on. This technology is even more relevant given the increasing complexity and dynamism of modern workplaces and operating environments. However, the vision for AI will only materialize if humans are able to successfully collaborate with these non-human partners. This is a difficult challenge as humans, on the one hand, do not fully understand how AI works and, on the other hand, are often already overburdened by the task (Lee and See, 2004; Hancock et al., 2011; Schaefer et al., 2016).

This challenge is even more critical in military domains, where warfighters are under increased (physical and cognitive) pressure and often face life and death situations (Kott and Alberts, 2017; TRADOC, 2018; Kott and Stump, 2019). Since the potential costs of failure are higher, warfighters tend to be even more reluctant to trust and collaborate with AI (Gillis, 2017). For these reasons, it is important to gradually increase exposure of AI technology to the warfighter through training that explains how AI works, its strengths, but also its limitations. Complementary, it is fundamental that, during mission execution, AI is capable of actively promoting trust and collaboration by communicating naturally, effectively, and efficiently with the warfighter, while minimizing the warfighter cognitive load.

Cognitive load is commonly defined as the difference between the cognitive demands of the task and the user’s available cognitive resources (e.g., attention and memory) (Hart and Wickens, 1990; Nachreiner, 1995; Mackay, 2000). Several factors have been identified that influence cognitive load but, broadly, it is possible to distinguish between exogenous factors (e.g., task difficulty) and endogenous factors (e.g., information processing capabilities for perceiving, planning, and decision making). Furthermore, individual factors, such as skill and experience, influence cognitive load. The importance of controlling cognitive load for task performance has been studied across several domains, with several studies suggesting that increased cognitive load can undermine performance (Cinaz et al., 2013; Dadashi et al., 2013; Vidulich and Tsang, 2014; Fallahi et al., 2016). More recently, there has been increasing interest on the impact that technology has on worker’s cognitive load. Much research has focused on the relationship between automation and user’s boredom and mental underload (Ryu and Myung, 2005), and its negative consequences on users’ ability to react in a timely manner during real or simulated flight (Shively et al., 1987; Battiste and Bortolussi, 1988), air combat (Bittner et al., 1989), air traffic control (Block et al., 2010), and so on. However, another concern is that technology may increase user’s cognitive load due to the change of routine processes, stress due to the transition, and general lack of understanding of how the technology works (Mackay, 2000).

Intelligent virtual assistants offer a promising route to promote collaboration between humans and AI, while controlling the impact on the user’s cognitive load (Gratch et al., 2002). Conversational assistants—i.e., intelligent virtual assistants that can communicate through natural language—have, in particular, been experiencing considerable commercial success—e.g., Apple Siri, Amazon Alexa, and Microsoft Cortana (Hoy, 2018). The basic premise is that advances in natural language processing technology (Hirschberg and Manning, 2015) enable more natural open-ended conversation with machines, which can then provide information or carry out users’ instructions. Verbal communication is also socially richer than other forms of communication like text or email, as it can convey pragmatic and affective information (Kiesler et al., 1984). Most current commercial systems, though, only have limited ability to convey this social richness through speech. Moreover, whereas natural communication is expected to improve productivity, the impact on users’ cognitive load from conversational assistants is still not well understood.

A fundamental limitation of conversational assistants, however, is their lack of embodiment and consequent limited ability to communicate non-verbally. Non-verbal communication plays an important role in regulating social interaction (Boone and Buck, 2003). Expressions of emotion, additionally, serve important social functions such as communicating one’s beliefs, desires, and intentions to others (Frijda and Mesquita, 1994; Keltner and Lerner, 2010; van Kleef et al., 2010; de Melo et al., 2014). The information conveyed by non-verbal cues, therefore, can be very important in building trust and promoting cooperation with humans (Bickmore and Cassell, 2001; Frank, 2004). Non-verbal communication in technology systems is typically achieved through robotic (Breazeal, 2003) or virtual agents (Gratch et al., 2002). Because these systems are embodied, they support non-verbal communication with users, including expression of emotion. Research shows that embodied conversational assistants can have positive effects in human–machine interaction (Beale and Creed, 2002; Beun et al., 2003; Kim et al., 2018b; Shamekhi et al., 2018), including building rapport (Gratch et al., 2006) and promoting cooperation with users (de Melo et al., 2011; de Melo and Terada, 2019, 2020). In the context of problem-solving tasks, pedagogical agents have been shown to be able to improve learning and task performance (Lester et al., 1997; Atkinson, 2002). These improvements are typically achieved through gestures that focus the user’s attention or through affective cues serving specific pedagogical purposes, such as motivating the user (Schroeder and Adesope, 2014). Embodied conversational assistants, therefore, hold the promise of having at least all the benefits in terms of task performance as (non-embodied) conversational assistants, while having minimal impact on the user’s cognitive load.

With increased immersion afforded to the user (Dey et al., 2018; Kim et al., 2018a), augmented reality (AR) has the potential to further enhance collaboration with AI. AR technology supports superimposition of virtual entities alongside the physical space in the user’s field of view. Therefore, first, interaction with AI systems can occur as the user is fully immersed in the task and, second, virtual interfaces—such as embodied assistants—can be integrated seamlessly in the workspace. Increased user influence in AR environments often occurs through an increased sense of social presence (Blascovich et al., 2002). Kim et al. (2018b, 2019) investigated the effects of an embodied conversational assistant on the sense of social presence and confidence in the system and found that both factors positively impacted users’ perception of the system’s ability to be aware and influence the real world, when compared to a (non-embodied) conversational assistant. Furthermore, participants perceived increased trust and competence in embodied assistants than the non-embodied counterparts. Wang et al. (2019) also conducted a study investigating user preference for different types of embodied or non-embodied assistants in AR while performing a visual search task together, and showed that participants preferred the miniature embodied assistant since the small size made assistants “more approachable and relatable.” A telepresence study, in contrast, suggested that participants were more influenced by an avatar, presumably representing another participant, that was the same size as a real person than by a miniature avatar (Walker et al., 2019); however, in this study, both types of avatar were located at a distance from the working environment and were not fully integrated in the task space. Overall, this research, thus, suggests that embodied conversational assistants in AR can be especially persuasive.

Here, we present an experiment where participants engaged in a relatively well-known collaborative problem-solving task—the desert survival task (Lafferty and Eady, 1974)—with an embodied assistant, a voice-only assistant, and no assistant. The task is implemented in an AR environment (Microsoft HoloLens). The assistants attempt to persuade participants with information pertinent to the task—e.g., “I heard the human body needs certain amount of salt for survival. Why don’t you move it up a bit?” They also provide general positive reinforcement (e.g., “You are doing great!”) and show appreciation when the participant follows the assistant’s suggestion (e.g., “Great! You listened to my suggestion.”). Embodied assistants further smile when making recommendations and, to focus the participant’s attention, move toward and point to the target of the suggestion. Our main hypothesis is that the embodied assistant will lead to increased task performance, when compared to the voice-only assistant (H1a); in turn, both assistants will lead to improved performance relative to the no assistant condition (H1b). We also look at participants’ subjective cognitive load and hypothesize that embodied assistants will lead to lower cognitive load, when compared to the voice-only assistant (H2). Finally, for the assistant conditions, we also look at measures of social presence and social richness (Lombard et al., 2009) and hypothesize that both will be perceived to be higher with embodied than voice-only assistants (H3). A preliminary report of these experimental results was also presented at the IEEE Conference on Virtual Reality and 3D User Interfaces (IEEE VR) 2020 (Kim et al., 2020).

## Experiment

### Methods

#### Design and Task

The experiment followed a within-participants design, with each participant engaging in the desert survival task with no assistant (control), a voice-only assistant, and an embodied assistant. The presentation order for the assistant conditions was counterbalanced across participants to minimize ordering effects. Based on the original version of the desert survival task (Lafferty and Eady, 1974), participants were given the following instructions: “You are in the Sonoran Desert, after a plane crash. You know you are 70 miles away from the nearest known habitation. You should prioritize the items on the table, by the importance of the item for your survival until you arrive there.” The participant is then asked to prioritize between 15 items such as bottle of water, jackknife, mirror, pistol, etc. This task has often been used in human–machine interaction studies (Morkes et al., 1998; Burgoon et al., 2000; Walker et al., 2019) for at least two reasons: first, since the solution is not obvious, it introduces an opportunity to test how persuasive a technology (e.g., a virtual assistant) is on the participant’s decision making; and, second, it has a clearly defined optimal solution^1^, which allows comparison of different technological solutions on task performance.

#### Assistant Suggestions

The assistants in the voice and embodied conditions were trying to help the participants make better decisions during the task by providing suggestions that could potentially improve the task score. The system recognized where the items were currently located during the task and calculated the current survival score continuously. In this way, the assistants could determine the item to suggest to move, such that the participants could make the largest improvement in the survival score if they followed the suggestion. The recommendation, thus, would always improve the score, with respect to the optimal solution, if followed. There were both positive and negative suggestions for each survival item, according to whether the suggestion was to move the item up or down in the ranking. For example, the positive suggestion for the flashlight was “The flashlight could be a quick and reliable night signaling device. Why don’t you move it up a bit?” and the negative suggestion was “I think the flashlight battery will be gone very quickly, so it might not be as useful as you expect. Why don’t you move it down a bit?” Table 1 shows the full list of positive and negative suggestions for all items. There were three different prompt variations for both moving up and down suggestions, e.g., “I think it’s ok to move it up/down,” and “I guess you can move it up/down more.” The assistant could also make stronger suggestions expressing that the item position should be adjusted a lot. For example, “I think you should move it up/down a lot,” “Why don’t you move it up/down a lot in the ranking?,” and “I’m sure you can move it up/down quite a lot.” The assistants could make the same suggestions repeatedly if the item was still the best option to improve the task score; however, if there was nothing to change for the score, no suggestion was provided. Participants received up to 10 suggestions from the assistant throughout the task. It is important to know that the assistants allowed the participants to decide whether they would follow the assistant’s suggestions or not; thus, if they wanted to, they could ignore the suggestion. After following suggestions, the assistants performed appreciation prompts, such as “Thank you for listening to my suggestion,” which could encourage more compliance by participants for follow-up suggestions. The assistant also gave general acknowledgment comments, which included some variations of simple assuring comments, such as “Okay,” “Good,” or “You are doing great so far.”

**TABLE 1:** Positive and negative suggestions for the items in the desert survival task.

**Item**	**Positive suggestion**	**Negative suggestion**
Air map	The air map might be helpful to check where you are heading and how much you should go further.	The air map might not be useful, since you already know the direction and the place where you have to go.
Book	You might need to hunt for food, so the book about edible animals seems useful.	Well, I guess the book about edible animals is not so useful. I think the problem could be dehydration, not starvation.
Coat	Well, I guess the top coat could be a good means, for keeping the moisture on your skin, and protecting you from hot and dry weather.	I guess the top coat might not be useful, with hot weather in the desert.
Compass	The compass would guide you the direction where you are heading to.	You can guess the direction, based on the Sun and the Moon. The compass might be of little use.
Compress	Well, I think the compress kit could be used as rope, or as a further protection against dehydration and sunlight.	The compass would guide you the direction where you are heading to.
Flashlight	The flashlight could be a quick and reliable night signaling device.	I think the flashlight battery will be gone very quickly, so it might not be as useful as you expect.
Jackknife	The jackknife would be useful for rigging the shelter, and for cutting up the cactus for moisture.	I’m not sure, but maybe the jackknife is not often used in the desert.
Mirror	I think the mirror could be quite useful to communicate your presence, by using the reflection of sunlight.	I’m not sure if the mirror is that important for your survival.
Parachute	The parachute might be useful as a shelter, or a signaling device, and it could also be used to make shade.	I guess the parachute might be burdensome, to bring all day, and I’m not sure how you would use it.
Pistol	I guess the pistol could be used as a sounding device, and it is good for protecting yourself from wild animals.	I guess there are not many dangerous creatures in the desert, so the pistol might not be that important.
Raincoat	I think the plastic raincoat could be useful, for protecting your body moisture, and it might even be used to extract some water, by the temperature differential.	It’s the desert. I’m not sure why you would need raincoats.
Salt	I heard the human body needs certain amount of salt for survival.	I guess the salt tablets would just require more body water to get rid of the increased salinity.
Sunglasses	The sunglasses would make eyes comfortable from the intense sunlight.	I guess the sunglasses are not that important. There should be many other ways to protect your eyes.
Vodka	I guess the vodka could be helpful for a fire, or as temporary coolant for the body.	I think the vodka might not be a good item to bring in the desert. Alcohol would absorb water.
Water Bottle	I think it would be good to drink the water, so you can remain clear-headed, when important decisions have to be made.	The water might not be that important. I guess there could be ways to easily get water in the desert, like cactus.

#### Voice-Only Assistant

Like other commercial systems (e.g., Alexa or Cortana), the assistant had a female voice. The speech prompts included task instructions, general acknowledgments, and the survival item suggestions. The assistant’s speech was also displayed in text as subtitles to the participant. Otherwise, the assistant had no visual cue throughout the task.

#### Embodied Assistant

**FIGURE 1:**
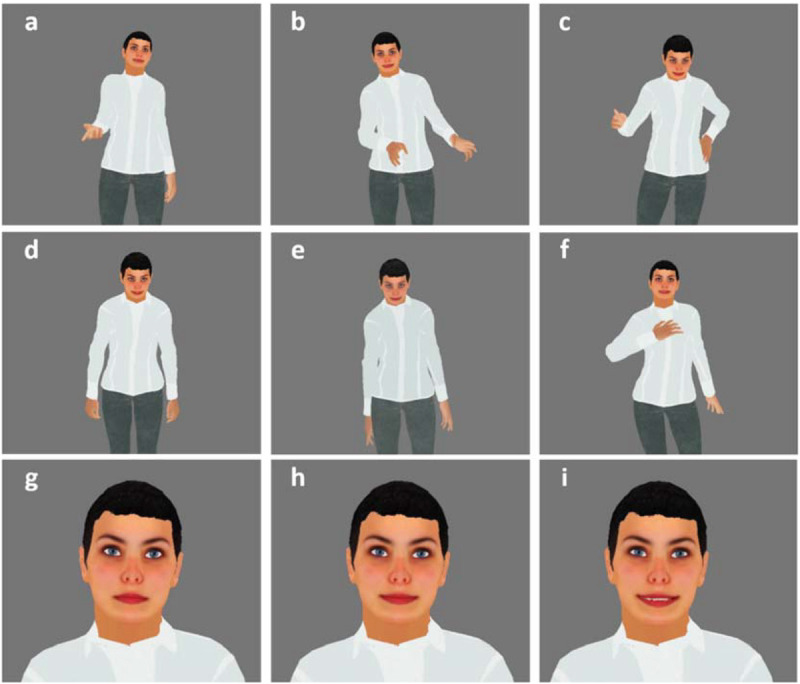
Embodied assistant gestures and facial expressions: **(a–c)** pointing gestures, **(d–f)** acknowledgment gestures, **(g)** neutral facial expression, **(h)** subtle smile, and **(i)** strong smile.

The embodied assistant was implemented as a female character, as shown in Figure 1. Building on prior work indicating a preference for miniature-sized characters (Wang et al., 2019), we made the assistant miniature-sized. This decision also meant that the assistant could move around the task space without forcing participants to avert their gaze. The assistant was animated with idle body motion and blinking and, when speaking, the lip motion was synchronized with the speech. When making a suggestion, the assistant would move next to the item in question and make a pointing gesture (Figures 1a–c). If the participant followed the suggestion, the assistant would also perform an acknowledgment gesture, such as a bow or putting the hand to the chest (Figures 1d–f). When making a suggestion or acknowledging, the assistant would gaze at the participant and show one of its smiling expressions (Figures 1h,i); otherwise, the facial expression would be neutral (Figure 1g).

#### AR Implementation

**FIGURE 2:**
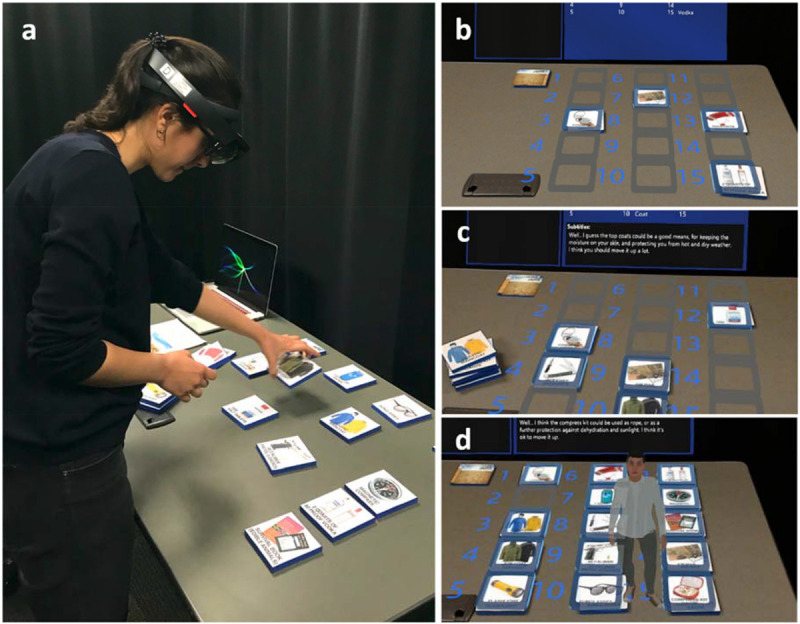
Desert survival task and assistant conditions: **(a)** participant’s physical space, **(b)** participant’s AR view for the no assistant (control) condition, **(c)** voice-only assistant condition, and **(d)** embodied assistant condition.

The AR environment for the desert survival task was implemented using Microsoft HoloLens technology and the Unity game engine. To complete the task, participants had to place real image markers illustrating the 15 survival items in the order of importance on a virtual board, while experiencing AR visual and auditory stimuli (Figure 2). The image markers were attached to physical foam bases so that the participants could intuitively grab them and move them around. To initiate the task, participants first looked at the marker with a desert image and put it on a start placeholder, which was virtually displayed on the table through the head-mounted display. Once the desert image marker was placed in the start placeholder, the instruction and state boards virtually appeared with 15-item placeholders on the table, where participants could place the survival items in their chosen order. When the item was placed in one of the placeholders, the placeholder turned to blue with a clicking sound effect and a virtual image corresponding to the item image was shown on it. Participants could freely re-order the items and check the status of placed items via a state board while performing the task. After all the 15 items were placed in the item placeholders, a finish placeholder was shown in AR next to the desert image marker, and the instruction guided the participants to put the desert marker on the finish placeholder to complete the task. Once participants placed the desert marker on the finish placeholder, the task was completed, showing a message that guided them to call the experimenter. The size of each marker was 10 cm × 10 cm × 1 cm. The PTC Vuforia Engine^2^ was used for marker recognition. The realistic voice for the assistants was pre-recorded using the Julie English voice from Vocalware’s text-to-speech^3^. The embodied assistant was a custom 3D model created using Adobe Fuse^4^ and FaceGen^5^. Body animations were created using Unity’s assets and inverse kinematics engine, as well as Mixamo^6^ animations. Please see the Supplementary Material for a video of the software.

#### Measures

The main measure was the score in the desert survival task. Score was calculated by summing the absolute differences between the participant’s ranking and the optimal ranking. To make the interpretation more intuitive, the sum of the differences was negated. This way, the best score was zero, which means all the items in the participant’s ranking match the optimal solution. The task performance gets worse as the score moves further along the negative scale.

To measure subjective cognitive load, we used the NASA Task Load Index (NASA-TLX) scale (Hart, 2006). Subjective scales are often used to measure cognitive load as they tend to be less intrusive than physiological scales (Meshkati et al., 1992). Even though several other subjective scales have been proposed (e.g., SWAT, Workload Profile), the NASA-TLX scale is still one of the most widely used and is sensitive to cognitive load, highly correlated with task performance, highly correlated with other subjective scales, but may be outperformed by other scales in task discrimination (Rubio et al., 2004). The NASA-TLX consists of six questions, each corresponding to one dimension of the perceived workload. For each dimension (mental demand, physical demand, temporal demand, performance, effort, and frustration), the participants provide a score on a scale, from “Very low” to “Very high,” consisting of 21 tick marks effectively identifying 5% delimitations on a scale of 0 to 100%. Participants then provide weights for each of the six dimensions via a series of binary choices to assess which dimensions were most important for the task; these weights are then factored into the final score by multiplying them with the dimension scores.

Regarding social presence, we adopted the social presence sub-scale from the Temple Presence Inventory (TPI) questionnaire (Lombard et al., 2009) and slightly modified it to assess participants’ sense of togetherness in the same space with the assistant, and the quality of the communication/interaction between them. The scale consists of seven questions on a seven-point scale (1, *not at all*, to 7, *very much*). We used this questionnaire only for a subjective comparison of the assistant conditions, i.e., the voice-only vs. embodied assistant conditions. For social richness, we adopted the social richness sub-scale from the TPI questionnaire (Lombard et al., 2009) to assess the extent to which the assistant is perceived as immediate, emotional, responsive, lively, personal, sensitive, and sociable. All the items for social richness are seven-point semantic differential scales (e.g., 1, *remote*, to 7, *immediate*). We also used this questionnaire only for the assistant conditions.

#### Procedure

Once participants arrived, they were guided to our laboratory space by an experimenter. They were asked to sit down in a room with a table and a laptop PC for answering questionnaires and were provided with the consent form. Once they agreed to participate in the experiment, they donned a HoloLens and went through the calibration procedure on the HoloLens to set their interpupillary distance. Afterward, participants had a practice session to learn how to use our marker-based interface by placing five animal markers. In this practice phase, they were asked to place the five animal markers in their preferred order on the table while experiencing AR visual feedback similar to the task. The experimenter was present next to the participants to answer any questions that they might have during the practice phase, while explaining the way to place and re-order the items. Once they felt comfortable with the marker-based interface, the experimenter described their actual task, the desert survival task, and the goal to prioritize the 15 items for their survival in a desert. In the description, participants were told that they were going to take part in the same task three times with some variations. Then, the first session started with one of the experimental conditions: either the control, the voice-only, or the embodied condition as described above. After completing the task, the participants were guided to complete several questionnaires measuring their perception of the experience in the desert survival task with or without assistant. When they were done answering the questionnaires, the experimenter guided them to repeat the same task in the next condition. Once the participants completed all three conditions, they answered further demographics and prior experience questionnaires, assessing their familiarity and experience with AR and virtual assistant technology. The participants were not informed about their performance on the survival task throughout the experiment. At the end, participants were provided with a monetary compensation ($15). The entire experiment took about an hour for each participant.

#### Sample

We recruited 37 participants from the University of Central Florida population for the experiment. Thirty-six participants completed the entire experiment, while one withdrew for personal reasons. We further excluded two more participants due to a failure to record data; thus, we had 34 participants (25 male and 9 female, ages 18 to 33, *M* = 21.90, *SD* = 4.10) for the analysis. All participants had normal or corrected-to-normal vision—12 with glasses and 7 with contact lenses. On a seven-point scale (from 1, *not familiar at all*, to 7, *very familiar*), the level of participant-reported familiarity with AR technology was comparatively high (*M* = 4.56, *SD* = 1.33). All participants had fewer than 10 previous AR head-mounted display experiences, and it was the first experience for 13 of them. Participants were also asked about their frequency of using commercial conversational assistant systems, such as Amazon Alexa, Apple Siri, or Microsoft Cortana. Their responses varied from no use at all to frequent daily use: eight participants indicated multiple times per day, two indicated once a day, eight indicated once a couple of days, seven indicated once a week, three indicated once a month, and six indicated no use at all. Five participants had prior experience with the desert survival task or closely related tasks.

### Results

**FIGURE 3:**
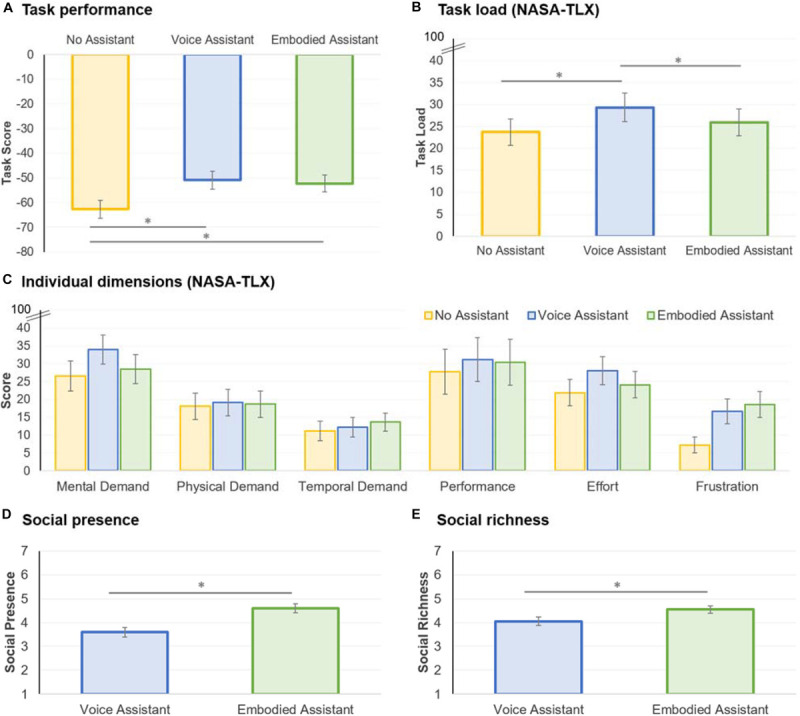
Task performance **(A)**, cognitive load **(B,C)**, social presence **(D)**, and social richness **(E)** experimental results. The error bars correspond to standard errors. ^∗^*p* < 0.05.

For task performance and subjective cognitive load, we ran a repeated-measures ANOVA and conducted *post hoc* comparisons with Bonferroni corrections. For cases where sphericity was not assumed, based on Mauchly’s test, we used the Greenhouse–Geisser correction if the Greenhouse–Geisser epsilon was lower than 0.750 or, otherwise, the Huynh–Feldt correction. For social presence and social richness, we conducted repeated measures *t* tests to compare the voice-only vs. embodied assistant scores. Figure 3 and Table 2 show the descriptive statistics for the experimental results.

**TABLE 2:** Means and standard errors for task performance, cognitive load, social presence, and social richness for all assistant conditions.

	**No assistant**		**Voice assistant**		**Embodied assistant**	
	**Mean**	***SE***	**Mean**	***SE***	**Mean**	***SE***
Performance	–62.71	3.67	–50.88	3.65	–52.29	3.40
Subjective cognitive load	23.73	3.03	29.37	3.26	25.94	3.06
Mental demand	26.62	4.25	33.97	4.10	28.53	4.07
Physical demand	18.09	3.74	19.12	3.67	18.68	3.73
Temporal demand	11.18	2.70	12.21	2.76	13.68	2.50
Performance	27.79	6.33	31.18	6.11	30.44	6.43
Effort	21.91	3.74	28.09	3.94	24.12	3.75
Frustration	7.21	2.24	16.62	3.47	18.53	3.61
Social presence	–	–	3.59	0.20	4.60	0.19
Social richness	–	–	4.05	0.18	4.55	0.16

#### Task Performance

The participants’ scores in the desert survival task are shown in Figure 3A. Recall that the optimal score is zero and that lower scores are worse. The analysis revealed a main effect for the assistant conditions, *F*(2,66) = 10.166, *p* < 0.001, $\eta_{\text{p}}^{2}$ = 0.236. *Post hoc* comparisons revealed that the score with no assistant was worse than with the voice-only (*p* = 0.002) and embodied assistants (*p* = 0.005); moreover, there was no statistically significant difference between the voice-only and embodied assistants (*p* = 1.000). The results suggest that assistants were able to improve participants’ performance, confirming hypothesis H1b; however, we found no support that embodied assistants improved performance when compared to voice-only assistants (hypothesis H1a).

Since we followed a within-participants design, we also wanted to get insight into any possible order effects. Accordingly, we ran a repeated-measures ANOVA on the task scores for the 1st, 2nd, and 3rd games. The results confirmed an order effect, *F*(1.405,66) = 16.029, *p* < 0.001, $\eta_{\text{p}}^{2}$ = 0.327: participants got the lowest score, averaged across all assistant conditions, on the 1st game (*M* = −63.35, *SD* = 19.974), followed by the 2nd game (*M* = −54.29, *SD* = 20.704), and, finally, the 3rd game (*M* = −48.24, *SD* = 20.986). This result suggests that there were learning effects with participants becoming more proficient at solving the task with each attempt, independently of the experienced assistant condition order. Nevertheless, the score pattern with the assistants in the 1st, 2nd, and 3rd games was the same as reported in the previous paragraph, though the effect was only statistically significant in the first game (1st game: *p* = 0.023, 2nd game: *p* = 0.168; 3rd game: *p* = 0.203).

#### Subjective Cognitive Load

Following the established method, the NASA-TLX scores were calculated by summing the weighted sub-dimension scores (Hart, 2006). The overall task load results are shown in Figure 3B and the dimensions are shown in Figure 3C. The analysis revealed a main effect for subjective cognitive load, *F*(1.631,66) = 5.243, *p* = 0.012, $\eta_{\text{p}}^{2}$ = 0.137. *Post hoc* comparisons confirmed that the voice-only assistant led to higher load than no assistant (*p* = 0.015) and embodied assistant conditions (*p* = 0.026). There was no statistical difference between the no assistant and embodied assistant conditions (*p* = 0.864). The result, thus, confirmed that embodied assistants led to lower cognitive load than voice-only assistants, in line with hypothesis H2. When looking at the NASA-TLX’s underlying dimensions, there was a trend for a main effect on mental demand—*F*(2,66) = 2.607, *p* = 0.081, $\eta_{\text{p}}^{2}$ = 0.073—and effort—*F*(2,66) = 2.020, *p* = 0.141, $\eta_{\text{p}}^{2}$ = 0.058—in line with the overall effect on cognitive load. In contrast, there was a main effect on frustration, *F*(2,66) = 8.230, *p* = 0.001, $\eta_{\text{p}}^{2}$ = 0.200, with participants reporting increased frustration with the voice-only (*p* = 0.016) and embodied assistants (*p* = 0.004) when compared to no assistant. This may have occurred because the recommendations were perceived as repetitive in some cases—for instance, one participant noted “the person slightly annoyed me, she kept repeating the same advice that I clearly did not care about”.

#### Social Presence and Social Richness

Regarding social presence, the analysis revealed a statistically significant difference between the assistants, *t*(33) = 4.568, *p* < 0.001, *d* = 0.622, with participants experiencing higher social presence with embodied than voice-only assistants (Figure 3D). The results also showed an effect on social richness, *t*(33) = 2.565, *p* = 0.015, *d* = 0.408, with participants experiencing higher social richness with embodied than voice-only assistants (Figure 3E). Thus, the results confirmed our hypothesis H3.

## General Discussion

As AI technology becomes pervasive in the modern workplace, teams consisting of humans and machines will become commonplace, but only if AI is able to successfully collaborate with humans. This requirement is even more critical in military domains, where warfighters are engaged in high-stakes, complex, and dynamic environments and, thus, under immense cognitive pressure (Kott and Alberts, 2017; Kott and Stump, 2019). Here, we present evidence suggesting that intelligent virtual assistant technology can be a solution for improving human task performance, while controlling cognitive load. Our experimental results indicate that, in an abstract problem-solving task, participants were able to produce higher-quality solutions when partnered with assistants, when compared to no assistants. Unlike our initial expectations though, embodied assistants did not improve performance over voice-only assistants. Prior research suggests that the ability to communicate non-verbally—e.g., bodily postures and facial expressions—can lead to increased rapport (Gratch et al., 2006) and cooperation (de Melo et al., 2011, 2014; de Melo and Terada, 2019, 2020) with users and, consequently, improved performance in collaborative tasks. In this case, though, it seems that the information communicated verbally had the most relevance for task performance, as suggested by some of the participants’ comments:

P4: “the assistant was very helpful, giving critical information in such a stressful situation if it happens in real world.” P7: “The interaction of the assistant was overall beneficial, as it brought up many things I wouldn’t have thought of.” P12: “The information provided by the assistant were great, it helped me prioritize items better.”

Interestingly, even though both assistants produced a bump in performance, the embodied assistant accomplished this with minimal impact on cognitive burden, as measured by the NASA-TLX scale, when compared to the voice-only assistant. This is particularly relevant given the attention that voice-only assistants—e.g., Amazon Alexa, Apple Siri, Microsoft Cortana—have been receiving (Hoy, 2018). Prior work with embodied assistants, especially in pedagogical applications, has been inconclusive about the impact of embodiment on cognitive load (Schroeder and Adesope, 2014): in some cases, post-tests were easier after interaction with an embodied agent (Moreno et al., 2001); in other cases, embodied agents led to increased cognitive load for learners, even though there was no impact on performance (Choi and Clark, 2006). Mayer’s multimedia learning theory can provide insight here (Mayer, 2001). Accordingly, there are two fundamental channels (auditory and visual) and optimal learning occurs when information is optimized across the channels—e.g., embodied agents would not produce an effect if they are redundant or irrelevant to the task (Craig et al., 2002). In our case, though, the embodied assistant was serving clear functions above and beyond the voice-only assistant: through facial expressions, it smiled when making suggestions; through its virtual body, it moved and pointed to the target of the suggestions; and, generally, through subtle cues (e.g., idle motion or blinking), the assistant conveyed a human-like presence in the task. The experimental results confirm that these kinds of non-verbal cues have meaningful impact on users’ subjective cognitive load. Participants’ comments, such as the one below, support the notion of a benefit of visual embodiment for helping participants feel more comfortable in collaborative situations with virtual assistants:

P28: “I like that the assistant is naturally in front of you and given at the same time as I worked rather than pausing just to listen to what she had to say.”

The results also showed that participants experienced higher social presence with embodied assistants than conversational assistants. Social presence relates to the ability of a communication medium to convey the sense that the user is immersed in the communication space and engaging in social interaction just as if it were face-to-face interaction (Short et al., 1976; Lowenthal, 2010). Research indicates that immersive technology—such as virtual or AR—have the potential to provide an increased sense of social presence, when compared to other media, such as phone or desktop (Blascovich et al., 2002). Our results, thus, support the idea that AR afforded increased immersion in the interaction with the embodied assistant, which may have contributed to reduced cognitive load. Related to the social presence effect, our results further indicate that participants perceived higher social richness with the embodied than the voice-only assistant. This suggests that people were more likely to treat interaction with the embodied assistant in a social manner, as if they were interacting with another human. This is in line with prior research indicating that increased human-like cues (Reeves and Nass, 1996) and immersion (Blascovich et al., 2002) can lead users to treat human–agent interaction like human–human interaction (Reeves and Nass, 1996; Mayer et al., 2003), which can lead to positive effects in terms of engagement, motivation, and interest (van Mulken et al., 1998; Atkinson, 2002; Moreno, 2005; Schroeder and Adesope, 2014). The social richness of the experience with the embodied assistant, thus, may have played a role in reducing the participants’ subjective cognitive load while performing the task.

The current work has limitations that introduce opportunities for future work. First, we have only explored simple emotion expression in the current work, with the assistant only showing various smiles throughout. However, research indicates that the social impact of emotion expressions depends on context (Hess and Hareli, 2016), and even smiles can lead to reduced cooperation when timed improperly (de Melo et al., 2014). Future work should consider more sophisticated emotion communication, which may lead to increased persuasiveness by the assistant (Shamekhi et al., 2018) and, ultimately, better performance. Second, our current speech synthesizer—like most commercial systems—has limited expressive ability. However, as speech technology improves, it will become possible to increase the bandwidth of multimodal expression (Gratch et al., 2002). Optimized multimodal expression can, then, lead to optimized transfer of information, learning, and performance (Mayer, 2008). Third, the current within-subjects design with a relatively small sample size could influence the participants’ performance and perception. Future work should complement the present work with between-subject designs. Still, when we compared the participants’ first trials as between-subjects comparisons, we found promising trends corresponding to our present results although not all the measures showed statistical significances, which encourages us to consider a further investigation in a between-subjects design with a large sample size. Fourth, we used Microsoft HoloLens in our experiment, but this was still the first generation of the prototype and, in practice, some participants still complained about the weight and bulkiness of the device. As AR head-mounted displays become better (e.g., lighter and supporting wider field of views), we can expect increased immersion and impact of embodied assistants. Sixth, we measured subjective cognitive load using the NASA-TLX scale; however, these findings should be complemented with physiological measures of cognitive load (Meshkati et al., 1992). Seventh, whereas the desert survival task captures many relevant aspects of collaborative problem solving, it is worth conducting further experimentation in more complex realistic tasks and attempt to replicate the effects reported here. Finally, the current prototype only implemented simple AI (e.g., to determine optimal suggestions and whether the participant followed suggestions), but it is possible to embed more intelligence and autonomy into assistant technology; the higher the autonomy, the more important is non-verbal behavior likely to be (Gratch et al., 2006; de Melo et al., 2011, 2014; de Melo and Terada, 2019).

Finally, the present work has important practical implications. The results confirm that assistant technology can improve task performance, if deployed appropriately. Even with a voice-only assistant, we were able to show a clear improvement in problem solving performance. Given the pace of evolution of natural language processing technology (Hirschberg and Manning, 2015), we can expect voice-only assistants to keep playing a pervasive and influential role. However, the results clearly indicate that voice-only assistants are fundamentally limited due to their inability to communicate non-verbally. Embodied assistants have the capability to engage users multimodally—like humans do (Gratch et al., 2002)—and complement the information conveyed through speech with appropriate gestures and emotion. Given increasing evidence of the important role of non-verbal and emotional expression in social interaction (Frijda and Mesquita, 1994; Boone and Buck, 2003; Keltner and Lerner, 2010; van Kleef et al., 2010; de Melo et al., 2014), developers and designers cannot afford to ignore the value of embodiment for assistant technology. Our results indicate that, by using non-verbal cues judiciously, we are able to control the users’ cognitive load while boosting performance, which is particularly critical when the stakes are high. This means that warfighters would be able to benefit from recommendations and actions of the assistant technology, while being able to focus their cognitive resources on other aspects of the task. Alternatively, this would support even more communication exchange and interaction with assistants, without overburdening the warfighter. The fast pace of development in AI technology and experimental research such as the one presented here clarifies how best to deploy this technology and introduces unique opportunities to create assistant technology that is immersive, feels like social interaction, is engaging and, most importantly, can promote optimal performance for the modern workforce and the warfighter in increasingly complex operating environments.

## Data Availability Statement

All datasets generated for this study are included in the article/Supplementary Material.

## Ethics Statement

All experimental methods were approved by the UCF Institutional Review Board (#FWA00000351 and IRB00001138).

## Author Contributions

CM and KK contributed to conception and design of the study, performed the statistical analysis, and wrote the first draft of the manuscript. KK and NN executed the study. All authors contributed to manuscript revision, and read and approved the submitted version.

## Conflict of Interest

The authors declare that the research was conducted in the absence of any commercial or financial relationships that could be construed as a potential conflict of interest.

## Footnotes

1 The optimal solution was based on the advice of Alonzo W. Pond, M.A., a survival expert who was a former chief of the Desert Branch of the Arctic, Desert, Tropic Information Center of the Air University at Maxwell Air Force Base.

2 https://developer.vuforia.com/

3 https://www.vocalware.com

4 https://www.adobe.com/products/fuse.html

5 https://facegen.com/

6 https://www.mixamo.com/
